# Somatosensory augmentation has sustained effects on mediolateral foot placement modulation while walking in people with chronic stroke

**DOI:** 10.1016/j.gaitpost.2025.110005

**Published:** 2025-10-08

**Authors:** Keith E. Howard, Ethan B. Schonhaut, Heather L. Knight, Camden J. Jacobs, Jesse C. Dean

**Affiliations:** a College of Health Professions, Medical University of South Carolina, Charleston, SC, USA; b Ralph H. Johnson VA Health Care System, Charleston, SC, USA

**Keywords:** Balance, Gait, Sensory augmentation Stroke, Vibration, Walking

## Abstract

**Background:**

Many people with chronic stroke (PwCS) exhibit reduced mediolateral foot placement modulation while walking, a deficit linked with poorer walking balance. Applying hip abductor vibration to augment the available somatosensory feedback can elicit immediate increases in foot placement modulation.

**Aim:**

The purpose of this study was to test whether such somatosensory augmentation can also cause sustained increases in foot placement modulation among PwCS after the vibration ceases, a beneficial characteristic for rehabilitation applications.

**Methods:**

44 PwCS performed a series of treadmill walking trials, with some trials including hip vibration designed either to augment sensory feedback (Augmentation group; n = 22), or to follow a random distribution (Noise group; n = 22). We quantified foot placement modulation using the partial correlation between foot placement and mediolateral pelvis displacement (ρ_FP_), accounting for pelvis velocity. ρ_FP_ was compared between a baseline walking period without vibration, periods when vibration was applied, and interspersed minute-long catch periods in which the vibration ceased.

**Results:**

In the Augmentation group, foot placement modulation increased significantly both while the vibration was applied (p < 0.0001) and during the subsequent catch periods (p = 0.007), providing evidence for short-term sustained effects of somatosensory augmentation. In contrast, no changes in foot placement were observed in the Noise group, either while the vibration was applied (p = 0.21) or during the catch periods (p = 0.36).

**Conclusion:**

These results provide initial evidence that somatosensory augmentation via hip vibration has effects on foot placement modulation that persist after the augmentation ceases, possibly due to sensory reweighting.

## 1. Introduction

Mediolateral foot placement modulation is an important strategy to ensure walking balance [1], but is often disrupted in people with chronic stroke (PwCS) [2]. In neurologically-intact individuals, foot placement scales with pelvis state, with more lateral foot placement when the pelvis is displaced farther mediolaterally from the stance foot [3]. This relationship is weakened or reversed in many PwCS; the partial correlation between pelvis displacement and foot placement (a measure of foot placement modulation) is lower in PwCS than neurologically-intact young controls walking at a similar speed by ~0.2 [4,5]. Beyond group differences, individual PwCS with lower foot placement modulation also tend to exhibit poorer performance on clinical balance measures [5].

The cause of reduced foot placement modulation in PwCS is unclear, due in part to multiple potential contributing factors. For example, an inability to accurately position the leg in the frontal plane can reduce foot placement accuracy [6] due to deficits in either sensorimotor accuracy or motor coordination. Alternatively, reduced foot placement modulation may be due to habitual use of a gait pattern learned early after the stroke – consistently placing the weaker paretic leg far laterally to avoid lateral losses of balance [7].

Several intervention strategies have targeted deficits in foot placement modulation. Most notably, foot placement can be prescribed using either static [8] or dynamic [9] visual cues. The latter approach has been applied to train walking “adaptability” - the ability to perform accurate stepping adjustments [10]. However, many PwCS do not respond in a timely fashion to mediolaterally shifting visual targets [11], perhaps due to perceived balance risks. Additionally, repeated training periods have not led to improved walking adaptability in comparison to more traditional treatment [12].

An alternative approach to improving foot placement modulation involves perturbing the swing leg. Pushing the swing leg away from an appropriate foot placement location (e.g., pushing the leg medially when lateral foot placement is warranted, and vice versa) elicits short-lived after-effects of increased modulation in both controls [13] and PwCS [14]. Repeated perturbation exposure caused improvements in foot placement modulation even after a 3-month follow-up period [7]. These results are consistent with error-driven sensorimotor learning, whereby perturbations increased movement errors and caused PwCS to adjust their foot placement control [7].

Rather than delivering mechanical perturbations, sensorimotor learning could conceivably be elicited using somatosensory augmentation, in which artificial sensory feedback improves users’ ability to perceive their body’s dynamic state [15]. For example, peripheral vibration can evoke proprioceptive feedback, which in turn alters the perception of the body’s mechanical state [16]. Even with the many neural changes that can occur after a stroke, most PwCS still exhibit predictable behavioral responses to peripheral vibration [17].

Our prior work leveraged the perceptual effects of vibration to elicit targeted behavioral responses. We demonstrated that appropriate vibration of the hip abductors increases foot placement modulation in controls [18], as abductor vibration can elicit a behavioral response consistent with the perception of the vibrated muscle lengthening [19–22]. Delivering vibration that scales with pelvis state can encourage either medial or lateral foot placement adjustments. This sensory augmentation approach also strengthens foot placement modulation in PwCS, although with more variable results than in controls [23].

While the positive effects of sensory augmentation on foot placement modulation are promising, the usefulness of this approach in rehabilitation is unknown. It is unclear whether increases in post-stroke foot placement modulation are sustained after augmentation ceases, as observed in controls [24]. This type of short-lived after-effect is often interpreted as an indicator of sensorimotor adaptation [25], which may contribute to longer-term learning with repeated exposure [26]. With sensory augmentation, such sustained effects may be caused by sensory reweighting [15]. Beyond potential sustained effects, it is unclear whether the positive effects of hip abductor vibration on foot placement modulation in PwCS are truly due to augmenting available sensory information or simply due to increased attention on the available somatosensory feedback [27].

The purpose of this study was to test whether somatosensory augmentation causes short-term sustained increases in foot placement modulation among PwCS. Participants performed multiple bouts of walking with hip vibration, separated by catch periods without vibration. We assessed the effects of two types of somatosensory stimulation: 1) Augmentation, in which vibration provides useful sensory information about the dynamic state of the body; 2) Noise, in which vibration is randomly delivered independent of the body’s dynamic state. We hypothesized that Augmentation stimulation would increase foot placement modulation while vibration is delivered, whereas Noise stimulation would not. Similarly, we hypothesized that Augmentation stimulation would cause positive sustained after-effects in foot placement modulation during catch periods without vibration, whereas Noise stimulation would not.

## 2. Methods

### 2.1. Participants

44 PwCS participated in this study. Inclusion criteria were: age ≥ 21 years; experience of a stroke ≥ 6 months prior; overground gait speed ≥ 0.2 m/s; ability to walk on a treadmill without a cane or walker. Exclusion criteria were: evidence of cerebellar damage; resting blood pressure > 220/110 mm Hg; history of cardiac medical issues; preexisting neurological issues or dementia; severe visual impairment; deep vein thrombosis or pulmonary embolism within 6 months; uncontrolled diabetes; orthopedic injuries or conditions in the lower extremities with the potential to alter the gait pattern; Botox injections in the hip musculature within 6 months. All participants provided written informed consent using a form approved by the Medical University of South Carolina Institutional Review Board.

Participants were randomized between two groups: Augmentation (n = 22) and Noise (n = 22). To justify this sample size, we conservatively assumed a medium effect size (Cohen’s d = 0.5), as the after-effects of vibration in PwCS may be smaller than in controls (Cohen’s d = 0.85) [24]. We performed a power analysis using Monte Carlo simulations of our linear mixed effects statistical model (detailed below), the assumed effect size, the distribution of our foot placement modulation metric among PwCS [5], and the correlation between this metric for paretic and non-paretic steps [5]. Based on this analysis, we found that our sample size would be sufficient to detect the assumed effect size with a power of over 80 %, assuming up to 10 % dropout or missing data, an alpha value of 0.05, a single after-effects period, and combining analysis of paretic and non-paretic steps. Study procedures were identical for assigned groups other than the hip abductor vibration (see Vibration Control section).

### 2.2. Experimental procedures

**Fig. 1.**
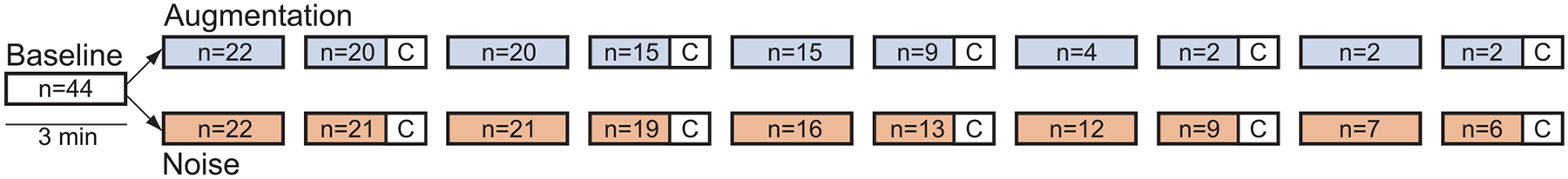
Sequence of 3-minute walking trials, progressing from left to right. After a baseline trial, participants were assigned to either the Augmentation (top row) or Noise (bottom row) group. Over the subsequent 10 trials, the assigned vibration type was delivered during periods indicated by shaded areas, with interspersed catch periods without vibration indicated as C. The number of participants (n) who completed each trial decreased over time due to fatigue.

Participants did not hold onto a handrail, but wore a harness attached to an overhead rail that would prevent falling to the ground. We identified participants’ self-selected treadmill walking speed by starting at 0.1 m/s and increasing in 0.05 m/s increments. When participants reported that the speed was faster than normal, speed was decreased to the previous level. Participants performed up to eleven 3-minute walking trials, with interspersed rest breaks. Rest breaks were at least 30-seconds, lasting until participants verbally reported being ready to continue. Trials ended if participants were too fatigued to continue or unable to maintain their treadmill position. A baseline trial involved no vibration (Fig. 1). In subsequent even-numbered trials, hip abductor vibration was applied for the entire trial. In odd-numbered trials, vibration was applied for the first 2-minutes but ceased in the final minute, a “catch period” to detect short-term sustained effects. Three participants fatigued before completing the first catch period and are not included in analyses. Table 1 provides demographic and baseline functional data for included participants.

### 2.3. Data collection and processing

LED markers (PhaseSpace; San Leandro, CA, USA) were secured over the sacrum and bilateral feet. Marker positions were sampled at 120 Hz and low-pass filtered at 10 Hz. We identified step starts when stepping foot velocity changed from posterior to anterior, and step ends when this velocity changed from anterior to posterior [5]. At each step start, we calculated the sacrum’s mediolateral displacement (relative to the stance heel) and velocity, with the positive direction toward the swing leg. At each step end, we calculated mediolateral foot placement as swing heel location relative to the sacrum.

Our primary outcome measure, ρ_FP_, quantified foot placement modulation; ρ_FP_ is the partial correlation between step start mediolateral pelvis displacement and step end mediolateral foot placement, accounting for step start mediolateral pelvis velocity. This metric was calculated separately for paretic and non-paretic steps, and for every minute (steps per minute: median=44, range=24–62). We chose this metric as our primary outcome measure because: 1) hip abductor sensory augmentation increases this metric in controls [18] and PwCS [23]; 2) sustained increases occur in controls after augmentation ceases [24]; and 3) this metric is related to clinical measures of walking balance and confidence in PwCS [5].

Secondarily, we used regressions to provide insight into the source of changes in ρ_FP_ [28,29]. For each participant, leg, and minute of walking, we regressed foot placement against mediolateral pelvis displacement. Metrics of interest were the slope between foot placement and pelvis displacement (β_disp_), average absolute error from the assumed linear relationship (ε), and standard deviations of foot placement (FP_var_) and pelvis displacement (Disp_var_). A stronger relationship between pelvis displacement and foot placement could be the result of a steeper slope, reduced error, or increased variance [29].

### 2.4. Vibration control

Vibrating tactors (EMS^2^; Engineering Acoustics Inc.; Casselberry, FL, USA) were secured over the bilateral hip abductors using silicone holders and elastic straps [18,23]. Tactor placement was standardized based on anatomical landmarks. Tactors were in direct contact with the skin, centered 2.5 cm anterior to the midway point between the hip greater trochanter and iliac crest (see Appendix A). These tactors deliver sinusoidal mechanical vibration with a maximum intensity of 74 Hz frequency and 0.95 mm peak-to-peak displacement, with higher intensities evoking stronger responses from primary muscle spindles [30].

Vibration intensity and location varied with each step, with a duration corresponding to the swing phase. In the Augmentation group, vibration was structured to strengthen mediolateral foot placement modulation. As previously described [18,23], mediolateral pelvis displacement was calculated at each step start, and compared to the participant’s distribution of baseline values. For displacements larger than the median, swing leg vibration was applied to encourage more lateral foot placement [22]. For displacements smaller than the median, stance leg vibration was applied to encourage more medial foot placement [19,22]. Vibration intensity scaled with the difference from the median displacement value, with justification for the scaling relationship in Appendix A.

In the Noise group, vibration location and intensity varied with each step, chosen randomly from a distribution mimicking that in the Augmentation group. These participants experienced the same type and intensity of abductor vibration, albeit without providing useful information about pelvis state.

### 2.5. Statistical analyses

Most participants (33 of 44) fatigued before the fourth catch trial. Therefore, our analyses included data only through the third catch trial, to reduce the risk of biasing results toward higher functioning participants. Steps with both legs were combined in our analyses, as prior work [23] observed no difference in the effects of augmentation between paretic and non-paretic steps. To investigate the immediate, direct effects of Augmentation vibration on ρ_FP_, we used a linear mixed-effects model with a fixed effects term for condition (Baseline vs. Augmentation) and random effects terms for stepping leg (paretic vs. non-paretic) and participant. To investigate the potential after-effects of Augmentation vibration, we used an identical model in which the fixed effects condition term compared Baseline and Catch periods. These analyses were repeated for the Noise group. The alpha value for statistical significance was 0.05. Identical analyses were conducted for the regression metrics (β_disp_, ε, FP_var_, and Disp_var_). Individual-level data are provided in Appendix B.

Following inspection of our primary results, we performed exploratory analyses to investigate potential changes in the effects of vibration on ρ_FP_ over time. For all periods with vibration, we used a linear mixed-model with a fixed effects term for walking time, and random effects terms for stepping leg and participant. Similarly, we used an identically structured linear mixed-model to investigate ρ_FP_ across all catch periods. These analyses were performed separately for the Augmentation and Noise groups.

## 3. Results

**Fig. 2.**
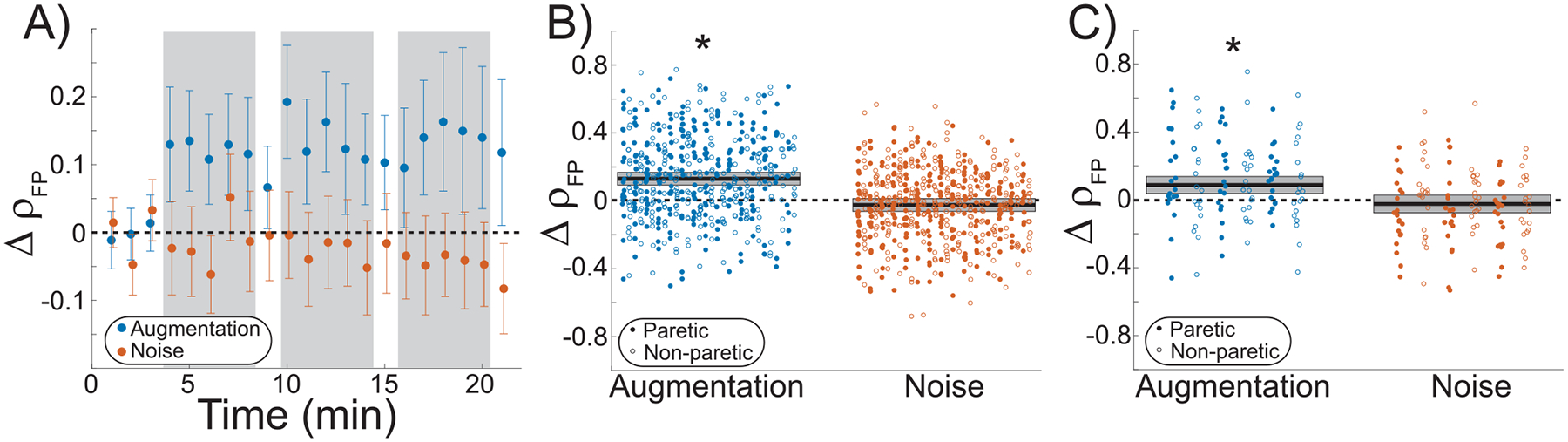
Effects of hip abductor vibration on foot placement modulation (ρ_FP_). A) The change in ρ_FP_ relative to the average baseline value is plotted for each minute of walking. Dots represent the mean change across participants who completed this minute of walking, and error bars represent 95 % confidence intervals. For simplicity, paretic and non-paretic values are combined. Shaded areas indicate times when vibration was applied. B) The direct effects of applying hip vibration are illustrated. Each dot indicates the ρ_FP_ value for an individual participant and minute. For illustrative purposes, paretic and non-paretic steps are plotted separately. Dots are clustered based on the minute of walking, with later minutes toward the right of the cluster. The dark horizontal line represents the estimated average effect, and the shaded area indicates the 95 % confidence interval. The asterisk (*) represents a significant change from baseline. C) Sustained effects of applying hip vibration are illustrated, plotting the change in ρ_FP_ relative to baseline for the three catch periods in which vibration was not applied. The structure of this panel parallels panel B.

Foot placement modulation was affected by vibration in the Augmentation group, but not the Noise group. Fig. 2A illustrates the average change in ρ_FP_ relative to baseline for each minute. Figs. 2B and 2C illustrate the variability in this change across participants. In the Augmentation group, ρ_FP_ was significantly greater during periods with vibration than during baseline (Table 2; Fig. 2B; p < 0.0001). In contrast, no significant difference in ρ_FP_ was observed between these periods in the Noise group (p = 0.21). Similar effects were present during the catch periods that followed bouts of vibration. In the Augmentation group, ρ_FP_ was significantly greater during catch periods than during baseline (Table 3; Fig. 2C; p = 0.0007). In the Noise group, ρ_FP_ did not differ significantly between these periods (Fig. 2C; p = 0.36).

Of the regression-based metrics, the only significant effects were observed in the Augmentation group for β_disp_, the slope between pelvis displacement and foot placement. Compared to baseline, this slope increased while vibration was delivered (Table 2; p < 0.0001) and during subsequent catch periods (Table 3; p = 0.002). No other significant changes were present (Tables 2–3).

In exploratory analyses, we found no statistical evidence that effects of vibration changed over time. In the Augmentation group, there was no significant effect of time on ρ_FP_ across vibration periods (p = 0.87; average slope = 0 [−0.004 0.003]; mean [95 % CI]), or catch periods (p = 0.41; average slope = 0.004 [−0.006 0.013]). Similarly, in the Control group, there was no significant effect of time on ρ_FP_ across vibration periods (p = 0.24; average slope = 0.002 [−0.005 0.001]), or catch periods (p = 0.22; average slope = 0.005 [−0.013 0.003]).

## 4. Discussion

Appropriately delivered hip abductor vibration increased foot placement modulation, a balance strategy often disrupted in PwCS. This modulation increased while vibration was applied and during subsequent catch periods, supporting our hypotheses. In contrast, foot placement modulation did not change when hip abductor vibration was unrelated to the ongoing body motion.

Hip abductor vibration increased foot placement modulation only when it augmented useful somatosensory feedback. In comparison to baseline, ρ_FP_ increased by an average of 0.13 with augmentation. This magnitude increase was larger than previously reported effects in both controls and PwCS (ρ_FP_ increase of ~0.05) [18,23]. In part, the larger effect in the present work is likely due to a higher gain between pelvis displacement and vibration intensity (Appendix A). The lack of an effect with noise vibration indicates that simply using vibration to increase attention on the hip abductors [27] was insufficient, consistent with prior work [18].

The simplest explanation for increased foot placement modulation during augmentation is that the vibration increases feedback magnitude through established sensory pathways [19–22]. This mechanism would not require a change in sensorimotor control strategy, as a constant relationship between sensory input and motor output could cause swing leg positioning to be more responsive to step-by-step fluctuations in pelvis state. Supporting this possibility, and consistent with results in controls [24], augmentation caused an increase in the slope between pelvis displacement and foot placement, but no change in other regression-based metrics. Additionally, a constant control strategy is consistent with the stable effects of augmentation over time, contrasting with adaptation to vibration often interpreted as feedback down-weighting [31].

However, increased foot placement modulation *after* augmentation ceases is not easily explained by a constant sensorimotor control strategy, which would cause a rapid return to baseline. After-effects were present in the Augmentation group but not the Noise group, and thus not due to treadmill walking accommodation [32] or increased attention on abductor feedback [27]. An alternative explanation for the short-term sustained effects of augmentation is sensory reweighting [15]. Augmented hip abductor feedback may allow more accurate perceptions of body dynamics, and linkage of these perceptions with other feedback sources (e.g., vestibular sense, foot cutaneous pressure) [33]. Once vibration ceases, users may continue to rely on these feedback sources (and their strengthened link with body state perception) to sustain increased foot placement modulation. Future work could test this potential mechanism by using sensory stimulation to assess changes in feedback gains (e.g., whether users become more responsive to galvanic vestibular stimulation after augmented somatosensation).

While promising, our results do not reveal the duration of sustained effects of augmentation on foot placement modulation. To have clinical relevance, a rehabilitation intervention would likely require multiple training sessions, and must elicit effects much longer lasting than those observed here. Such information is generally lacking for sensory augmentation [15], although a small-scale study using vibrotactile cueing during standing balance exercises reported sustained beneficial effects 6 months after training [33]. In terms of foot placement modulation, a novel perturbation method that elicited short-lived (~1-minute) beneficial after-effects with a single exposure [13,14] caused sustained improvements (12 weeks post-intervention) with repeated exposure [7]. The sustained increases in foot placement modulation observed in the present study thus motivate future investigation of the effects of longer-term augmentation exposure.

Several additional limitations should be noted. The sensorimotor effects of vibration are variable [17,34], influenced by vibration location and intensity as well as individual participant differences of unknown etiology. Our approach may thus only be useful for a subset of PwCS. Additionally, we focused on the effects of augmenting hip abductor feedback, whereas numerous other sources of sensory feedback can also influence foot placement [35–37]. Perhaps multisensory augmentation would have larger effects than any form of unisensory augmentation. Finally, despite randomization, the median time since stroke was notably (albeit not significantly; Wilcoxon rank-sum test, p = 0.16) longer in the Noise group (76 months) than the Augmentation group (37 months). While it’s feasible that the longer chronicity in the Noise group could have caused greater opportunity for recovery [38] or functional deterioration due to reduced activity levels [39], similarities in clinical test scores between groups indicate this was not the case.

In conclusion, hip abductor vibration designed to augment somatosensory feedback caused both immediate and short-term sustained increases in foot placement modulation, an important strategy for walking balance. While these results are consistent with beneficial sensory reweighting, future work is needed to assess this potential mechanism and provide insight into the clinical usefulness of this approach.

## Supplementary Material

1

2

## Figures and Tables

**Table 1:** Group demographic and baseline functional characteristics. All numerical values are presented as median [range].

	Augmentation (n = 20)	Noise (n = 21)
Gender	6 F / 14 M	6 F / 15 M
Paretic side	12 L / 8 R	11 L / 10 R
Age (yrs)	64.5 [34–80]	57 [35–81]
Height (cm)	174 [157–185]	169 [152–192]
Mass (kg)	80 [62–115]	87 [56–116]
Time since stroke (mo)	37 [9–169]	76 [7–282]
FM motor score	25.5 [17–34]	24 [11–30]
FGA	16 [5–26]	16 [8–23]
ABC	72 [31–99]	69 [21–99]
Treadmill speed (m/s)	0.4 [0.15–1.1]	0.4 [0.15–1.15]

**Table 2:** Effects on gait metrics while vibration is delivered. Values are presented as the model estimated change from baseline, and the 95 % confidence interval of this change in brackets. Significant changes (p < 0.05) are indicated by bolding.

Metric	Augmentation		Noise	
	Δ from baseline	P-value	Δ from baseline	P-value
ρ_FP_	**0.13 [0.09 0.17]**	**< 0.0001**	−0.02 [−0.06 0.01]	0.21
β_disp_	**0.17 [0.12 0.22]**	**< 0.0001**	−0.05 [−0.10 0.00]	0.06
ε(mm)	−0.1 [−0.5 0.3]	0.60	−0.2 [−0.6 0.1]	0.22
FP_var_ (mm)	−0.3 [−0.8 0.2]	0.24	−0.1 [−0.6 0.4]	0.57
Disp_var_ (mm)	0.2 [−0.2 0.5]	0.34	0.0 [−0.4 0.4]	0.95

**Table 3:** Effects on gait metrics during catch periods after vibration is delivered. Values are again presented as the model estimated change from baseline, and the 95 % confidence interval of this change in brackets. Significant changes (p < 0.05) are indicated by bolding.

Metric	Augmentation		Noise	
	Δ from baseline	P-value	Δ from baseline	P-value
ρ_FP_	**0.09 [0.04 0.14]**	**0.0007**	−0.02 [−0.08 0.03]	0.36
β_disp_	**0.11 [0.04 0.18]**	**0.002**	−0.05 [−0.12 0.02]	0.17
ε(mm)	0.1 [−0.5 0.6]	0.84	0.0 [−0.5 0.4]	0.88
FP_var_ (mm)	−0.2 [−1.0 0.6]	0.63	0.3 [−0.4 1.0]	0.44
Disp_var_ (mm)	0.1 [−0.4 0.6]	0.76	0.2 [−0.3 0.7]	0.38

## Appendix A. Supporting information

Supplementary data associated with this article can be found in the online version at doi:10.1016/j.gaitpost.2025.110005.
