# Supplementary material for: Somatosensory augmentation has sustained effects on mediolateral foot placement modulation while walking in people with chronic stroke
Source: Gait Posture. Author manuscript; Available in PMC 2026 Jun 23. (PMC13289792; doi:10.1016/j.gaitpost.2025.110005)
Supplement: 1 [file NIHMS2184629-supplement-1.docx]

**Somatosensory augmentation has sustained effects on mediolateral foot placement modulation while walking in people with chronic stroke**

**Appendix A**

**Introduction.** Prior work has demonstrated that mediolateral foot placement modulation can be increased by somatosensory augmentation in the form of targeted hip abductor vibration among both neurologically-intact controls (Knapp et al., 2021; van Leeuwen et al., 2024) and people with chronic stroke (PwCS) (Schonhaut et al., 2024). Specifically, foot placement modulation increased when vibration was applied to the stance hip for steps in which the pelvis was close to the stance foot (encouraging more medial foot placement) and applied to the swing hip for steps in which the pelvis was far from the stance foot (encouraging more lateral foot placement). In these studies, the control relationship between pelvis displacement and vibration intensity was chosen based on intuition and small-scale pilot data rather than formal testing. Therefore, the primary goal of this preliminary experiment was to determine whether the gain of the control relationship between pelvis displacement and vibration intensity influenced foot placement modulation.

Secondarily, this experiment sought to determine whether the location of the applied vibration over the hip abductors affected foot placement modulation. Our prior work has targeted the gluteus medius (the largest primary hip abductor muscle) by placing the vibrating tactors directly superior to the greater trochanter, at the midway point between the greater trochanter and the iliac crest (Flack et al., 2012). However, the gluteus medius has a fan-shaped structure, widening as it travels superiorly (Flack et al., 2014). Further, this muscle can be subdivided into distinct compartments based on fascicle orientation and innervation with slightly different mechanical actions, and thus potentially different somatosensory feedback. The most common proposed division of this muscle is into anterior, middle, and posterior components (Al-Hayani, 2009; Flack et al., 2014).

| **Metric** | **Value** |
| --- | --- |
| Gender | 12 F / 30 M |
| Paretic side | 18 L / 24 R |
| Age (yrs) | 61.5 [37-83] |
| Height (cm) | 173 [152-192] |
| Mass (kg) | 83.5 [56-115] |
| Time since stroke (mo) | 67.5 [9-271] |
| FM motor score | 26 [11-33] |
| FGA | 18 [4-27] |
| ABC | 73 [38-99] |
| Treadmill speed (m/s) | 0.45 [0.2-1.05] |

**Methods.** 42 PwCS participated in this experiment, with basic demographic and functional characteristics provided in Table S1. The inclusion and exclusion criteria were the same as for the experiment described in the main text, and all participants provided informed consent using a form approved by the Medical University of South Carolina Institutional Review Board.

For all trials, participants walked on a treadmill at their self-selected speed, identified using the same process described in the main text. An initial 2-minute walking trial without vibration was used to identify each participant’s distribution of mediolateral pelvis displacement values at the start of each step taken with the paretic or non-paretic legs. Participants then performed a series of nine 2-minute walking trials in randomized order. These nine trials corresponded to every combination of three vibration gains (High, Medium, or Low) and three vibration locations (Anterior, Middle, and Posterior).

**Table S1.** Demographic and baseline functional characteristics. All numerical values are presented as median [range].


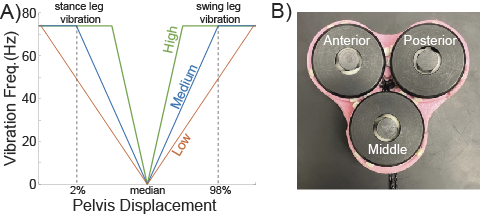
Vibration gain was defined as the slope of the relationship between pelvis displacement and vibration frequency, as illustrated in Figure S1A. Briefly, with higher gains, participants would experience maximum intensity vibration in a larger proportion of steps. Vibration location was controlled using a custom silicone holder that secured tactors over the anterior, middle, and posterior aspects of the gluteus medius (Fig. S1B).

**Figure S1.** Vibration control parameters. A) The gain of the relationship between pelvis displacement at the start of each step and vibration frequency was varied. As in prior work, each participant’s distribution of pelvis displacement values for steps taken with each leg was quantified at the start of the session during a walking trial without vibration. The median, 2^nd^ percentile, and 98^th^ percentile values of this distribution were identified. In subsequent trials, steps that started with a pelvis displacement less than the median were accompanied by stance leg vibration, while steps with a pelvis displacement greater than the median were accompanied by swing leg vibration. The frequency of this vibration varied across gain conditions. For the Medium gain condition used in our prior work, the frequency increased as the pelvis displacement deviated more from the median value, reaching its maximum for all pelvis displacement values less than the 2^nd^ percentile or greater than the 98^th^ percentile. For the High gain condition, the slope of this relationship was doubled. For the Low gain condition, the slope of this relationship was reduced by one third, as pictured. B) Vibration location was varied using silicone holders (pictured for the right hip) that mimicked the anatomical shape of the gluteus medius, with tactor centers separated by 5 cm.

As in the main text, markers placed over the sacrum and bilateral feet were used to identify the start and end of steps, and to quantify our primary outcome measure (ρ_FP_) for paretic and non-paretic steps. We used a two-way ANOVA with interactions to determine whether vibration gain or location influenced ρ_FP_. These statistical comparisons were performed separately for paretic and non-paretic steps. In the event of significant effects, post-hoc Tukey-Kramer tests were performed to compare gain or location conditions, as appropriate. The alpha value was set to 0.05 for all comparisons.

**Results.** Mediolateral foot placement modulation was influenced by vibration gain, but not vibration location. Specifically, for steps taken with the paretic leg, gain had a significant (p=0.008) effect on ρ_FP_, with the lowest foot placement modulation for the Low gain (Fig. S2A). In contrast, location (p=0.29) and the interaction between gain and location (p=0.55) did not significantly affect foot placement modulation. Similar effects were observed for steps taken with the non-paretic leg, as gain significantly (p=0.032) influenced ρ_FP_ (Fig. S2B), with the High gain causing greater modulation than the Low gain. Again, ρ_FP_ was not significantly affected by location (p=0.21) or the interaction between gain and location (p=0.24). Across all nine tested gains and locations, the largest magnitude paretic foot placement modulation was observed with a High gain and Anterior location.


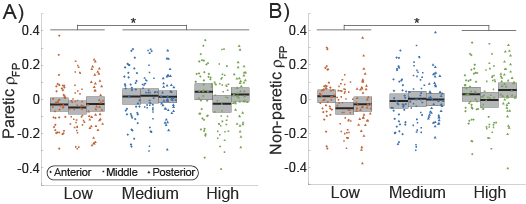
**Discussion.** The gain of the control relationship used to determine the intensity of vibration influenced foot placement modulation. Essentially, the Low gain condition was less likely to cause large pelvis displacements to be accompanied by lateral foot placement and small pelvis displacements to be accompanied by medial foot placement. The simplest explanation for this observation is that higher intensity vibration is more likely to evoke a behavioral response (Eklund, 1972), and the low gain condition delivered vibration with lower intensity. However, the present results also suggest an upper limit on the effectiveness of increasing the augmentation gain, as foot placement modulation did not differ significantly between the Medium and High gain conditions. This observation may be attributable to characteristics of the specific vibration control system used here, as the vibrating tactors’ maximum intensity was reached for many (~32%) steps in the High gain condition. This ceiling effect placed an upper limit on the dynamic range of somatosensory feedback that participants could experience.

**Figure S2.** Foot placement modulation (ρ_FP_) values across tactor gains (Low, Medium, High), and locations (Anterior, Middle, Posterior). Markers represent individual participant values for each of the nine conditions for paretic (A) and non-paretic (B) steps. Horizontal lines represent mean values, and shaded areas represent 95% confidence intervals. For illustrative purposes and to focus on differences between conditions rather than between participants, data values are plotted as the difference from each individual’s mean value across all conditions. Asterisks indicate significant post-hoc differences between the indicated gain conditions.

Unlike vibration gain, vibration location did not have a significant effect on foot placement modulation. These results thus do not provide direct support for the idea that the complex anatomical structure of the gluteus medius will cause artificial sensory feedback from different regions of the muscle to have different behavioral effects. We acknowledge that despite the lack of a statistically significant effect, the magnitude of the effects elicited by vibration delivered to the Middle location appeared smaller than the Anterior or Posterior locations. While speculative, it is possible that this may be due to the structure of our silicone holder, in which the Middle tactor location was also more inferior. Although we ensured that all tactors were over the muscle belly, there may be less muscle mass on average in this region, as the gluteus medius narrows as it runs inferiorly (Flack et al., 2014), which may have contributed to this smaller magnitude effect.

**References**

Al-Hayani, A., 2009. The functional anatomy of hip abductors. Folia Morphol (Warsz) 68, 98–103.

Eklund, G., 1972. General features of vibration-induced effects on balance. Ups. J. Med. Sci. 77, 112–124.

Flack, N. a. m. s., Nicholson, H. d., Woodley, S. j., 2014. The anatomy of the hip abductor muscles. Clinical Anatomy 27, 241–253. https://doi.org/10.1002/ca.22248

Flack, N.A.M.S., Nicholson, H.D., Woodley, S.J., 2012. A review of the anatomy of the hip abductor muscles, gluteus medius, gluteus minimus, and tensor fascia lata. Clinical Anatomy 25, 697–708. https://doi.org/10.1002/ca.22004

Knapp, H.A., Sobolewski, B.A., Dean, J.C., 2021. Augmented Hip Proprioception Influences Mediolateral Foot Placement During Walking. IEEE Trans Neural Syst Rehabil Eng 29, 2017–2026. https://doi.org/10.1109/TNSRE.2021.3114991

Schonhaut, E.B., Howard, K.E., Jacobs, C.J., Knight, H.L., Chesnutt, A.N., Dean, J.C., 2024. Altered foot placement modulation with somatosensory stimulation in people with chronic stroke. J Biomech 166, 112043. https://doi.org/10.1016/j.jbiomech.2024.112043

van Leeuwen, A.M., Bruijn, S.M., Dean, J.C., 2024. Force-field perturbations and muscle vibration strengthen stability-related foot placement responses during steady-state gait in healthy adults. Hum Mov Sci 96, 103243. https://doi.org/10.1016/j.humov.2024.103243
